# Exploring Chromogranin A (CgA) as a Diagnostic Marker in Hypothermia-Related Deaths: Two Case Studies and a Literature Review

**DOI:** 10.3390/diagnostics15131673

**Published:** 2025-06-30

**Authors:** Luca Tomassini, Erika Buratti, Giulia Ricchezze, Roberto Scendoni

**Affiliations:** 1 International School of Advanced Studies, University of Camerino, 62032 Camerino, Italy; luca.tomassini@unicam.it; 2 Department of Law, Institute of Legal Medicine, University of Macerata, 62100 Macerata, Italy; g.ricchezze1@unimc.it (G.R.); r.scendoni@unimc.it (R.S.)

**Keywords:** hypothermia, immunohistochemistry, chromogranin A, neuroendocrine activation, forensic pathology

## Abstract

**Background:** Hypothermia, occurring when core temperature drops below 35 °C, can lead to death when the body’s heat loss exceeds its heat production. This study investigates two hypothermia-related deaths, exploring the utility of immunohistochemistry, specifically focusing on chromogranin A (CgA) as a potential diagnostic tool. The aim is to assess whether CgA expression in neuroendocrine tissues can be considered a reliable indicator of premortem stress response in fatal hypothermia cases. **Case Presentation:** In the first case, a 67-year-old man was found on a snowy road 24 h after his disappearance. The autopsy revealed cold-induced skin lesions, gastric hemorrhages, and cerebral and pulmonary edema. Positive CgA immunostaining was observed in the pancreatic islets and adrenal medulla. In the second case, a 49-year-old man was found dead in a wooded area with indications of suicide. Both cases were examined with attention to macroscopic findings and histological samples from major neuroendocrine organs. As in previous cases, CgA immunostaining was positive in the pancreatic islets and adrenal medulla. Staining intensity was moderate to strong, consistent with heightened neuroendocrine activity, supporting the hypothesis of systemic stress prior to death. **Conclusions:** Although CgA is a potentially valuable adjunct in hypothermia diagnosis, careful consideration of cadaveric preservation is emphasized, particularly when bodies are preserved before autopsy. Further studies with larger sample sizes are needed to confirm its diagnostic specificity and to distinguish true pathological patterns from postmortem artifacts.

## 1. Introduction

Hypothermia is defined as a drop in core body temperature below 35 °C, and it occurs when the body’s heat loss surpasses its heat production [1]. It is a leading cause of mortality associated with exposure to extreme environments, such as mountainous or marine settings [2]. In these cases, it is referred to as exogenous hypothermia, as distinct from endogenous hypothermia and hypothyroidism-induced hypothermia [3]. However, hypothermia has also been reported in elderly and/or debilitated individuals, under certain circumstances within their own homes, involving both exogenous and endogenous factors [3]. Typically, in response to body cooling, a series of endocrine and metabolic changes corresponding to the range of low temperatures occur. Mortality from hypothermia has been reported at over 70% for body core temperatures of 30 °C and over 90% for body core temperatures of 26 °C [4,5].

The pathophysiology of hypothermia involves a progressive depression of central nervous system activity, respiratory rate, cardiac output, and renal perfusion. In response to cold stress, the hypothalamus initiates thermoregulatory mechanisms including vasoconstriction, shivering, and hormonal activation. These mechanisms become impaired as core temperature drops, eventually leading to cardiac arrhythmias, unconsciousness, and death [6]. From a forensic standpoint, identifying hypothermia as the cause of death is often difficult due to the non-specific nature of external and internal findings. This challenge has led to increasing interest in postmortem biomarkers and tissue-based diagnostics.

Immunohistochemistry (IHC) is considered a potentially valuable technique for diagnosing hypothermia. Notably, chromogranin A (CgA), although not yet validated as a reliable adrenal biomarker, has shown significant utility at the hypothalamic level, suggesting persistent neuroendocrine activation during hypothermic states [7,8,9,10]. 

CgA is a glycoprotein stored and released from neuroendocrine granules along with catecholamines, making it a promising indicator of stress-related physiological states. Studies have shown that CgA immunoreactivity increases in regions such as the hypothalamus, adrenal medulla, and pancreatic islets during hypothermic conditions, reflecting the systemic activation of the sympathoadrenal system in response to cold exposure [9]. Moreover, various studies suggest that combining CgA expression patterns with classical autopsy signs may enhance diagnostic specificity in ambiguous forensic scenarios, especially when hypothermia overlaps with other stress-related fatal events. This reinforces the potential of CgA as a supportive tool in the forensic diagnosis of fatal hypothermia.

This study presents two cases of hypothermia-related deaths in which immunohistochemistry was performed, and accompanying images are provided.

## 2. Case Presentation

### 2.1. Case 1

A 67-year-old man voluntarily left his home by car in February. Approximately 24 h after his disappearance, investigators tragically found his corpse on a snowy country road; the vehicle was found about 1 km away. His niece informed investigators that the man was cardiopathic and had suffered a prior stroke and that, as a result, he reported recurrent transient global amnesia episodes. At the time of discovery, meteorological records indicated ambient temperatures ranging between 2 °C and −1 °C. 

#### 2.1.1. Autopsy

Following its discovery, the body was transported under cold chain conditions and stored at approximately 4 °C for 12 h prior to autopsy. Temperature monitoring confirmed stable refrigerated conditions throughout, minimizing autolytic changes and preserving tissue antigenicity for immunohistochemical evaluation. At the time of autopsy, the body was well-preserved, with no evidence of advanced decomposition or postmortem damage, ensuring the reliability of both histological and immunohistochemical evaluations. External examination revealed lesions consistent with cold exposure. In particular, intense hypothermia in both legs and some abrasions on the dorsum of the left hand were found. Notably, there were signs of pronounced hypothermic effects in both lower limbs and abrasions on the dorsum of the left hand. A high degree of bright red hypostasis was observed.

Internal examination revealed several degenerative pathological alterations, primarily affecting the central nervous system. These include cerebral atrophy in the left occipital lobe, which is the site of a previous stroke, and widespread cerebral atherosclerosis. Signs of hypertensive heart disease were also highlighted. Examination of the coronary tree, assessed through serial sections, revealed overall vessel patency with signs of coronary atherosclerosis, in the absence of significant stenosis. Chronic pulmonary emphysema was observed in the apical regions of the lungs. In addition, acute pathological findings related to the events under investigation were present, including pinpoint perivascular brain hemorrhages, numerous small hemorrhagic erosions on the gastric mucosa (Wischnewsky spots) (WSs), diffuse cerebral edema, pulmonary edema, and significant polyvisceral congestion. Toxicological investigations yielded negative results.

#### 2.1.2. Histopathology

Tissue samples from the adrenal medulla and pancreatic islets were formalin-fixed and paraffin-embedded (FFPE) and then sectioned at approximately 4 µm and mounted on positively charged slides. Immunohistochemical staining was performed using the BenchMark ULTRA^®^ automated staining system (Ventana Medical Systems, Inc., Roche Diagnostics, Ancona, Italy) in combination with the anti-chromogranin A (LK2H10) primary antibody (Ventana™, Cat. No. 760-2519) [11]. For antigen retrieval, ULTRA Cell Conditioning Solution (ULTRA CC1) was applied at 100 °C for 24 min. The primary antibody was incubated for 4 min at 36 °C. Detection was carried out using the OptiView DAB IHC Detection Kit (Ventana™, Cat. No. 760-700), followed by counterstaining with Hematoxylin II for 4 min and Bluing Reagent for 4 min.

Standard hematoxylin and eosin (H&E) staining was also performed on all tissue sections prior to immunohistochemical analysis, in order to evaluate the general histoarchitecture and identify background pathological changes. 

**Figure 1:**
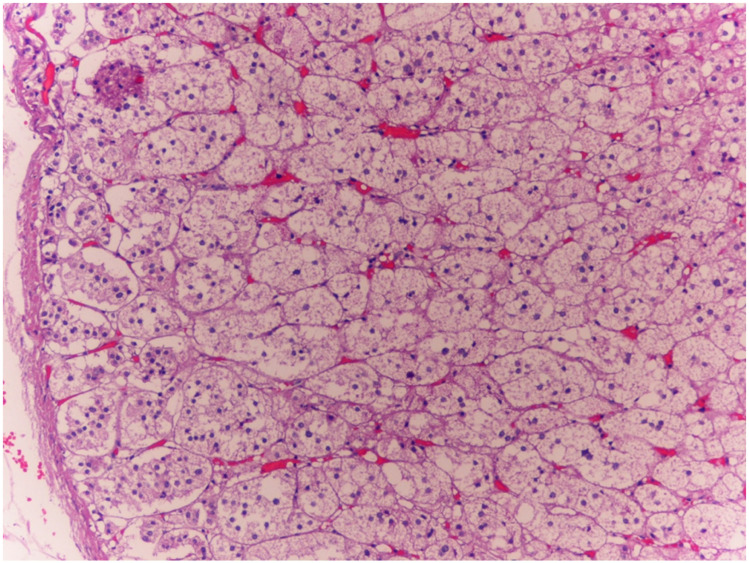
Case 1: In the adrenal glands, glandular cells had a distinctive spongy appearance, accompanied by an almost complete loss of typical intracellular lipid droplets (10×). Within the cortical region of the adrenal glands, lipid droplets were barely present, a finding consistent with the hypothesis that the mechanism leading to the demise of both subjects examined in this study was not inherently acute. This observation aligns with the explanations provided by certain experts who have studied the topic of adrenal postmortem examinations [10,12].

The histological examinations confirmed the macroscopic examination of organs; petechial brain injury was documented, along with cerebral edema with aspects of intracytoplasmic vacuolation and visceral congestion. Microscopic examination of the myocardium revealed mild perivascular and interstitial fibrosis. Steatosis (fatty liver) was found. In the adrenal glands, a characteristic spongiform appearance of the cortical cells was observed, marked by an almost complete loss of normal intracellular lipid droplets. (Figure 1). Finally, the structural aspect of the endocrine pancreas was of particular importance as it showed marked atrophy of the islets of Langerhans, widely filled by vacuoles, and the presence of microbleeds.

**Figure 2:**
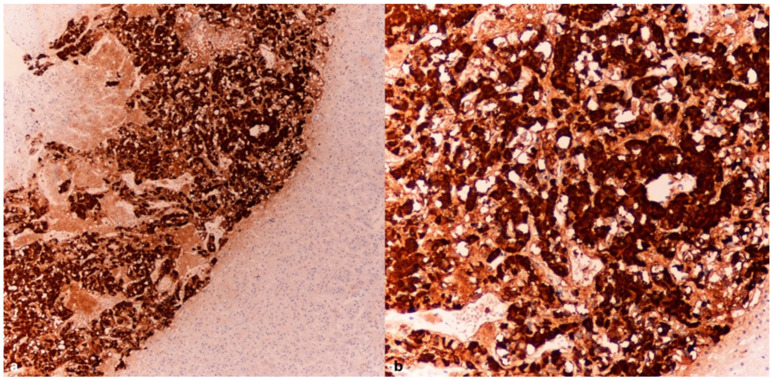
Case 1: Adrenal medulla–CgA tissue samples (4× (**a**), 10× (**b**)) were obtained from the adrenal glands of a subject who succumbed to hypothermia, revealing a pronounced expression of chromogranin A (CgA).

**Figure 3:**
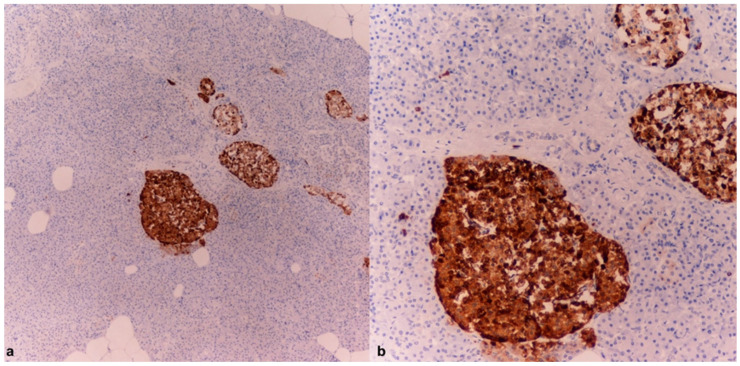
Case 1: Pancreatic tissue with distinct evidence of islets of Langerhans strongly positive for chromogranin A (4× (**a**), 10× (**b**)).

The immunohistochemical investigations for CgA revealed an intense positivity in the cells of the adrenal medulla (Figure 2a,b) and the islets of Langerhans (Figure 3a,b).

### 2.2. Case 2

A 49-year-old man was tragically discovered deceased within a secluded wooded area. The decedent had a documented twenty-year history of battling depression and had previously undergone coronary artery bypass surgery for a heart condition. According to acquaintances and family, he had never expressed suicidal ideation. At the scene, the body was discovered wearing only a pair of boxer shorts, which were notably soiled with earthy material, and the right gluteal region presented several small lacerations. His remaining articles of clothing were found scattered nearby. A handwritten letter was recovered from the inner pocket of a jacket located close to the body; its contents clearly articulated the individual’s intention to engage in self-harm. Environmental data from the local meteorological station reported nighttime temperatures fluctuating between 3 °C and 1 °C.

#### 2.2.1. Autopsy

Upon retrieval, the corpse was stored in a controlled mortuary environment at 4 °C for 18 h prior to autopsy. Storage temperature logs indicated continuous refrigeration without interruption.

**Figure 4:**
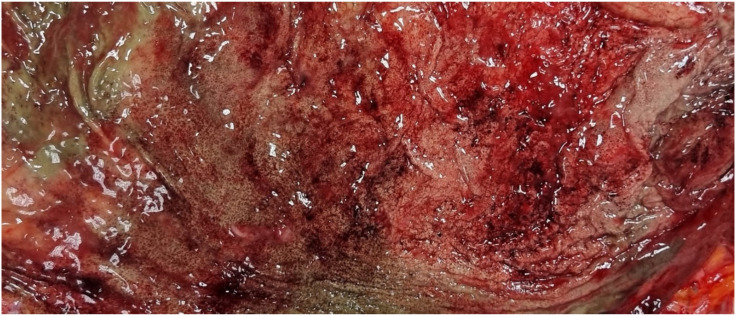
Case 2: Wischnewski spots represent a well-established indication of hypothermia in postmortem examinations, yet their occurrence in living individuals is exceedingly rare. The pathogenesis has been linked to disruptions in microcirculation, ischemic events, and reperfusion injuries [13].

The body exhibited signs of partial rigor mortis and freezing. External examination revealed a median sternotomy scar consistent with a prior cardiac surgical procedure, along with postmortem lesions consistent with scavenging activity by wild animals. There was evidence of coronary artery bypass grafting, with complete patency of the graft between the aorta and the right coronary artery; the accessible coronary segments showed no significant atherosclerotic changes. Additionally, there were long and numerous red-brown streaks in various parts of the body, with a higher concentration in the gluteal region. Internal examination revealed good preservation of organs, remnants of double coronary artery bypass, mild chronic pulmonary emphysema, and modest steatotic hepatomegaly. Cerebral perivascular hemorrhages, superficial hemorrhagic erosions of the gastric mucosa (WSs) (Figure 4), and cerebral and pulmonary edema were also observed. Toxicological investigations yielded negative results.

Despite environmental exposure, the body showed excellent preservation, with well-maintained internal structures, which enabled accurate tissue sampling and immunohistochemical evaluation.

#### 2.2.2. Histopathology

Tissue from the adrenal medulla and pancreatic islets was formalin-fixed, paraffin-embedded (FFPE), and sectioned at 4 µm. Immunohistochemistry was performed on a BenchMark ULTRA^®^ system (Ventana/Roche) using the anti-chromogranin A (LK2H10) antibody (Ventana™, 760-2519). Antigen retrieval was carried out with ULTRA CC1 (24 min, 100 °C), followed by 4 min primary antibody incubation at 36 °C. Detection used the OptiView DAB kit (Ventana™, 760-700), with Hematoxylin II and Bluing Reagent as counterstains. Routine H&E staining was performed for morphological assessment.

**Figure 5:**
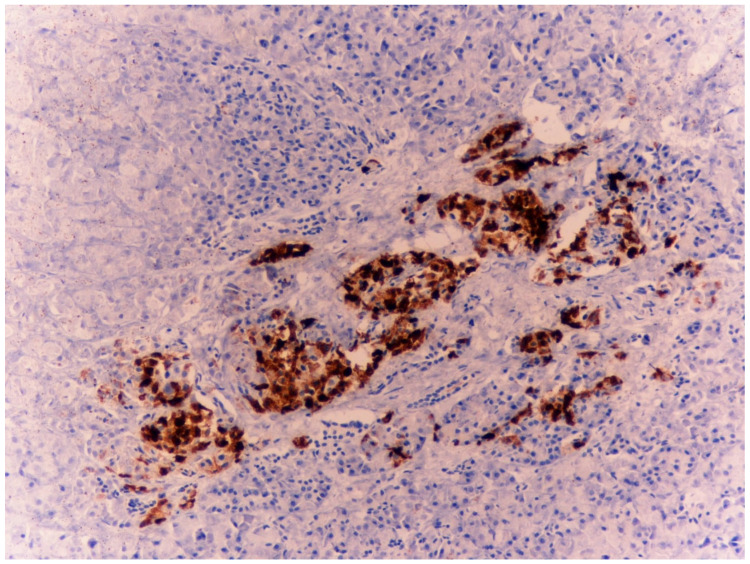
Case 2: Three pancreatic tissues with evidence of islets of Langerhans strongly positive for chromogranin A—4×.

The histological examinations confirmed macroscopic observations, including small perivascular hemorrhages in the brain. Microscopic examination of the myocardium revealed interstitial and perivascular fibrosis, with a more prominent area of fibrous replacement in the posterior wall of the left ventricle, without evidence of acute pathology. Dilation of the cutaneous blood vessels, aspects of hepatic steatosis, moderate fibrosis, steatosis, and myocardiosclerosis were observed. In the pancreas, features of chronic pancreatitis with intense cytoplasmic eosinophilia in acinar exocrine cells and vacuolization of the islets of Langerhans were noted. Immunohistochemical investigations for CgA highlighted intense positivity in the cells of the adrenal medulla and islets of Langerhans (Figure 5).

**Figure 6:**
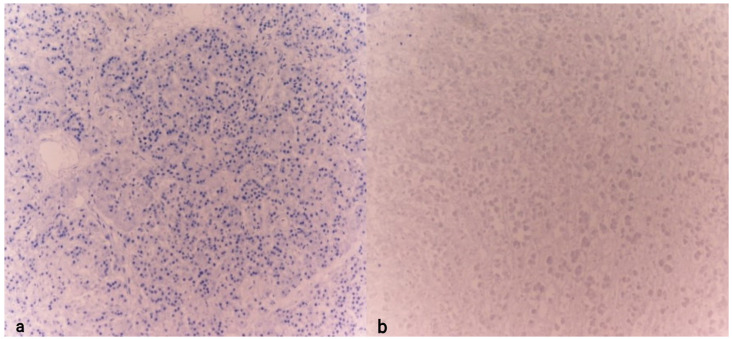
Detail of the pancreas (**a**) and adrenal gland (**b**) examined for CgA in individuals with sudden death. Note the absence of marker expression (10× (**a**), 10× (**b**)).

Both cases were compared with control subjects whose cause of death could not be identified as hypothermia (sudden cardiac death and instantaneous death following a road traffic accident). In the control cases, tissue samples were collected following a timeline and under postmortem storage conditions similar to those of the previously described cases. Notably, the samples from the control cases tested negative for chromogranin A and showed a markedly poorer state of tissue preservation (Figure 6).

## 3. Discussion

This study investigated the immunohistochemical expression of chromogranin A (CgA) in two postmortem cases of suspected hypothermia. Both individuals were found in cold environments, and autopsies revealed findings consistent with fatal cold exposure, including Wischnewski spots (WSs), cerebral and pulmonary edema, and peripheral lesions. Immunohistochemical analysis revealed strong CgA positivity in the adrenal medulla and pancreatic islets in both cases. These results suggest activation of the neuroendocrine system in response to terminal cold stress.

The strong expression of CgA in the adrenal medulla and pancreatic islets in both individuals supports the hypothesis of a systemic neuroendocrine activation as a terminal physiological response to hypothermia. This is consistent with the role of CgA as a co-stored protein with catecholamines in chromaffin granules and endocrine tissues. The absence of hypothalamic evaluation in these cases limits more detailed neuroanatomical interpretation, but the findings indicate an increased endocrine activity aimed at maintaining homeostasis during agonal stress.

Notably, both subjects showed remarkably similar clinical and environmental contexts, including ambient temperatures between −1 °C and 3 °C, external lesions consistent with hypothermia, cerebral and pulmonary edema, and strong chromogranin A (CgA) positivity in the adrenal medulla and pancreatic islets of Langerhans. These findings suggest a comparable degree of environmental stress. In this context, CgA expression may reflect a pre-terminal physiological effort to adapt to prolonged cold exposure. Additionally, in Case 1, the adrenal cortex showed a spongy appearance with marked lipid depletion—an important finding that may indicate prolonged activation of the hypothalamic–pituitary–adrenal (HPA) axis. This pattern supports a sustained stress response prior to death, consistent with a non-instantaneous hypothermic process. It reinforces the interpretation of CgA expression as part of a broader neuroendocrine reaction to cold-induced stress. Table 1 summarizes the main clinical, autopsy, and immunohistochemical findings in the two cases.

Chromogranin A is a soluble acidic glycoprotein highly expressed in neuroendocrine cells and chromaffin vesicles [12,13]. In recent years, numerous studies have highlighted its emerging role as a biomarker in heart failure, metabolic disorder, tumor biomarker, and postmortem biochemical analyses [4,9,10,14,15,16,17,18]. Its ability to predict mortality in heart failure patients has been described in various studies, including those conducted by Ceconi et al., which demonstrated a strong correlation between plasma CgA levels and disease severity, suggesting its use in clinical risk stratification [19]. Furthermore, biochemical studies on chromaffin vesicles have revealed CgA’s involvement in secretory granule dynamics [20]. Freezing and thawing experiments have shown changes in the redistribution of lipid phosphates, dopamine-β-hydroxylase, and CgA, highlighting its role in vesicle stabilization and catecholamine secretion regulation [21]. Recent evidence also suggests that CgA is involved in metabolic regulation [22]. Research by Bandyopadhyay et al. has shed light on its influence on peripheral insulin sensitivity and lipid metabolism, opening new perspectives on understanding obesity and metabolic dysfunctions [23].

Increasing attention has been directed toward the role of CgA-derived peptides, such as catestatin and vasostatin, which appear to exert regulatory effects on cardiovascular homeostasis, inflammation, and immune responses [24,25]. Catestatin, in particular, has been shown to modulate cardiac inotropy and vasodilation, as well as to inhibit sympathetic nerve activity, thus contributing to cardiovascular protection in conditions of stress and heart failure [26]. These findings support the broader clinical utility of CgA and its fragments not only as markers but also as potential therapeutic targets.

In addition to its impact on cardiology and metabolism, CgA is gaining increasing relevance in forensic sciences. Some studies have investigated its use in postmortem biochemistry, showing that CgA immunohistochemistry can provide crucial information on fatal stress conditions, including hypothermia and hyperthermia [8,27]. This ability to serve as an indicator of extreme stress makes CgA a potentially useful tool in forensic medicine.

In forensic pathology, such biochemical alterations may serve as temporal markers of physiological reactions occurring during the perimortem period, particularly in cases where traditional morphological indicators are inconclusive or absent.

Yoshida et al. (2009) [9] reported increased CgA expression in specific tissues (adenohypophysis, adrenal medulla, pancreatic islets) in hypothermia cases, aligning with our findings. Immunohistochemical analysis demonstrated CgA overexpression in the cells of the adenohypophysis, adrenal medulla, and pancreatic islets (approximately 50–80%), compared to other similar glands.

More recently, Tomassini et al. (2025) observed a reduced hypothalamic expression of CgA in hypothermia-related deaths, possibly reflecting suppressed neurosecretory function due to prolonged cold [28]. However, preserved positivity in the adrenal medulla and pancreatic islets, as observed in the two cases presented in this study, supports the hypothesis of a generalized endocrine response.

According to Yoshida et al. [9], CgA expression in the adenohypophysis and adrenal medulla did not significantly differ among various causes of death, suggesting a more generalized stress response. A positive correlation was also observed between hypothalamic CgA expression and cerebrospinal fluid (CSF) levels in hypothermia cases—an association not seen in the other two sites.

Moreover, circulating CgA levels have been shown to rise under critical illness and acute stress, further reinforcing CgA’s utility as a biochemical indicator of perimortem physiological adaptation [19].

Given its multifactorial role, it is evident that chromogranin A represents a versatile biomarker with broad implications in cardiology, metabolism, and forensic medicine. Its ability to reflect various pathological states underscores the importance of further research to better define its clinical and diagnostic applications.

This study presents several limitations that should be acknowledged. First, the absence of core body temperature data at the time of discovery limited the ability to quantitatively correlate the immunohistochemical findings with the actual hypothermic state. Furthermore, postmortem changes—such as ischemia, hypoxia, and tissue degradation—may have affected antigen preservation and, consequently, chromogranin A (CgA) expression. Histological interpretation may also have been compromised by artifacts related to refrigeration and tissue handling. Another limitation was the lack of hypothalamic assessment, which prevented a comprehensive evaluation of central neuroendocrine responses. Finally, although CgA immunostaining was evident in stress-responsive tissues, its expression is not pathognomonic and should be interpreted carefully, always in conjunction with classical morphological features and circumstantial evidence.

The ability of CgA to reflect systemic neuroendocrine activation highlights its potential as a complementary postmortem marker in forensic medicine. Specifically, CgA immunopositivity in the adrenal medulla and pancreatic islets may indicate a premortem stress response, useful when traditional signs are equivocal or absent.

When combined with classical signs—such as Wischnewski spots, petechiae, and peripheral vasodilation—CgA staining may improve diagnostic confidence in hypothermia cases. Importantly, its expression may also help distinguish between sudden death and deaths following prolonged physiological distress, with possible medico-legal relevance [29].

However, given its lack of diagnostic specificity and susceptibility to postmortem variability, CgA should not be used in isolation, but rather integrated into a broader forensic assessment.

## 4. Conclusions

The findings of this study support the potential role of chromogranin A (CgA) as a supportive immunohistochemical marker in the postmortem evaluation of hypothermia. The strong CgA expression observed in the adrenal medulla and pancreatic islets in both cases suggests a systemic endocrine activation in response to terminal cold-induced stress. These results are consistent with the existing literature and reinforce the view of CgA as a context-dependent indicator of premortem physiological stress.

Although CgA and other endocrine markers offer promising avenues for postmortem investigation of hypothermia, their forensic applicability remains limited by several methodological and interpretive challenges. These include the lack of pathognomonic staining patterns, interindividual variability in tissue response, and the influence of postmortem intervals, tissue degradation, and preservation conditions. Proper cadaver handling and preservation prior to autopsy are essential to avoid misinterpreting normal postmortem changes as hypothermia-specific findings.

Immunohistochemistry for CgA is a practical and relatively accessible technique in forensic pathology, capable of yielding valuable insights when used in conjunction with traditional autopsy findings and circumstantial data. However, to enhance its diagnostic reliability, standardization of sampling, fixation, and staining protocols is needed. Future studies involving larger case series, multiple organs, and correlation with core body temperature and survival intervals are warranted to clarify the diagnostic utility and specificity of CgA in hypothermia-related deaths.

In this context, while CgA cannot yet be considered a definitive marker, it may serve as a useful complementary tool in the forensic assessment of suspected hypothermia, particularly in cases with ambiguous or inconclusive findings.

## Figures and Tables

**Table 1:** Comparative summary of clinical, histological, and immunohistochemical findings in the two hypothermia-related cases.

	Available Data of the Cases	
Parameter	Case 1	Case 2
Age	67 years	49 years
Location found	Snowy country road	Secluded wooded area
Ambient temperature (°C)	−1 to 2 °C	1 to 3 °C
Time to autopsy	12 h refrigerated	18 h refrigerated
Key autopsy findings	WSs, cerebral/pulmonary edema, petechiae	WSs, cerebral/pulmonary edema, dermal vessel dilation
CgA adrenal medulla	Strong positivity	Strong positivity
CgA pancreatic islets	Strong positivity	Strong positivity
CgA hypothalamus	Not assessed	Not assessed

## Data Availability

The authors confirm that the data supporting the findings of this study are available within the article.

## Abbreviations

The following abbreviations are used in this manuscript:

CgA	Chromogranin A
HPA	Hypothalamic–pituitary–adrenal
IHC	Immunohistochemistry
WSs	Wischnewsky spots
